# Lower versus higher oxygen targets for out-of-hospital cardiac arrest: a systematic review and meta-analysis

**DOI:** 10.1186/s13054-023-04684-3

**Published:** 2023-10-19

**Authors:** Xin Cheng, Yu Zhang, Haidong Deng, Yuning Feng, Weelic Chong, Yang Hai, Pengfei Hao, Jialing He, Tiangui Li, Liyuan Peng, Peng Wang, Yangchun Xiao, Fang Fang

**Affiliations:** 1 West China Hospital, Sichuan University, No. 37, Guo Xue Xiang, Chengdu, 610041 Sichuan China; 2 Affiliated Hospital of Chengdu University, Chengdu, Sichuan China; 3 Thomas Jefferson University, Philadelphia, PA USA

**Keywords:** Out-of-hospital cardiac arrest, Oxygen target, Mortality

## Abstract

**Background:**

Supplemental oxygen is commonly administered to patients after out-of-hospital cardiac arrest. However, the findings from studies on oxygen targeting for out-of-hospital cardiac arrest are inconclusive. Thus, we conducted a systematic review and meta-analysis to evaluate the impact of lower oxygen target compared with higher oxygen target on patients after out-of-hospital cardiac arrest.

**Methods:**

We searched the Cochrane Central Register of Controlled Trials, MEDLINE, Embase, from inception to February 6, 2023, for randomized controlled trials comparing lower and higher oxygen target in adults (aged ≥ 18 years) after out-of-hospital cardiac arrest. We screened studies and extracted data independently. The primary outcome was mortality at 90 days after cardiac arrest. We assessed quality of evidence using the grading of recommendations assessment, development, and evaluation approach. This study was registered with PROSPERO, number CRD42023409368.

**Results:**

The analysis included 7 randomized controlled trials with a total of 1451 participants. Compared with lower oxygen target, the use of a higher oxygen target was not associated with a higher mortality rate (relative risk 0.97, 95% confidence intervals 0.82 to 1.14; I^2^ = 25%). Findings were robust to trial sequential, subgroup, and sensitivity analysis.

**Conclusion:**

Lower oxygen target did not reduce the mortality compared with higher oxygen target in patients after out-of-hospital cardiac arrest.

**Supplementary Information:**

The online version contains supplementary material available at 10.1186/s13054-023-04684-3.

## Background

Out-of-hospital cardiac arrest (OHCA) is defined as the loss of functional cardiac mechanical activity in association with an absence of systemic circulation, occurring outside of a hospital setting [1]. OHCA is a leading cause of global mortality [1], and hypoxic ischemic encephalopathy is the main cause of disability and mortality in patients after OHCA [2]. Supplemental oxygen is commonly administered to patients after OHCA, aiming to prevent hypoxemia. There are two distinct oxygen targets: lower target and higher target. The guidelines in 2015 and 2017 recommend the administration of high inspired oxygen for patients with resuscitation following OHCA [3, 4]. However, high inspired oxygen levels can potentially be harmful [5], as excessive oxygen intake can lead to adverse effects such as lung injury, decreased cardiac output, decreased local blood flow, inflammatory cytokine production and free radicals generation [4, 6–10]. Hence, the optimal oxygen target for patients after OHCA remains a topic of debate.

Several previous meta-analyses examining the impact of lower and higher oxygen targets on patients after OHCA have presented inconsistent findings. Young et al. reported a reduction in mortality associated with lower oxygen target [11], while Holmberg et al. found no statistically significant difference [12]. However, these previous meta-analyses were primarily limited by small sample sizes, with trials including only a limited number of participants. Recent publication of two large-scale trials on this topic have yielded significant supplementary data, effectively bolstering the sample size [13, 14]. Therefore, a new analysis incorporating these trials is warranted.

To confirm the oxygen target, we conducted a systematic review aiming to compare the effects of lower and higher oxygen targets in patients after OHCA.

## Methods

### Protocol and guidance

This systematic review was conducted according to the Preferred Reporting Items for Systematic Reviews and Meta-Analyses (PRISMA). The protocol for the current study was prospectively submitted to the International Prospective Register of Systematic Reviews (PROSPERO) (ID: registration number: CRD42020152179).

### Selection criteria

Studies were included if they (1) enrolled OHCA adults (aged ≥ 18 years); (2) compared higher and lower oxygen targets, measured by any one of the following: fraction of inspired oxygen, arterial partial pressure of oxygen, arterial oxygen saturation (measured by blood analysis), or peripheral oxygen saturation; (3) reported outcome of interest; (4) were randomized controlled trials (including individually randomized trials, cluster randomized trials, quasi-randomized trials). Studies were excluded if they were cross-over randomized trails.

### Outcomes

The primary outcome was mortality at 90 days. Mortality at 30 days or mortality in hospital was used to compute the pooled analysis if mortality at 90 days was not reported.

Secondary outcomes included length of hospital stay (days, measured as hospital discharge date minus date of emergency department admission, including both survivors and non-survivors), neuron‑specific enolase (NSE, a serum marker of neuronal injury during the early post-resuscitation period in humans) at 48 h, favorable modified Rankin scale score (0–2; mRS, ranging from 0 to 6, with higher scores indicating greater disability) at the last reported time point, and favorable Cerebral Performance Category (0–2; CPC, with higher values indicating more severe disability) at 90 days.

### Information sources and search strategy

We searched the Cochrane Central Register of Controlled Trials, MEDLINE, Embase, from inception to February 6, 2023. No language restrictions were applied. The details of search terms are demonstrated in Additional file 5: Table S1.

### Study selection

Two reviewers (HD and XC) independently screened all titles and abstracts retrieved by the systematic search. Disagreements were resolved by discussion or adjudicated by a third reviewer (YZ). Two reviewers then reviewed the articles retained for full-text assessment. Disagreements regarding eligibility were resolved by discussion.

### Data extraction

Two reviewers (HD and XC) independently extracted data on the characteristics of the included trials, including details such as the study region, study population, study design, number of participants, mean age and intervention specifics. To ensure accuracy, a third reviewer (YZ) checked for any errors in the extracted data. Disagreements between reviewers were resolved by discussion.

### Assessment of risk of bias

Two reviewers (HD and XC) independently assessed the risk of bias of trials using the Cochrane Risk of Bias tool across seven domains [15]. Each domain in all trials was assigned a study-level score indicating the level of bias risk: low, high, or unclear. Disagreements between reviewers were resolved by discussion. A final judgment was provided by a third author (YZ) if consensus could not be reached.

### Confidence of evidence

Two authors (HD and XC) independently assessed the quality of evidence for primary and secondary outcomes using the Grading of Recommendation, Assessment, Development and Evaluation (GRADE). The quality of evidence was categorized as high, moderate, low, or very low based on multiple factors including the evaluation of study design, risk of bias, inconsistency, imprecision and indirectness of the included trials [16].

### Data analysis

We conducted the statistical analysis using RevMan (version 5.3, The Cochrane Collaboration). For dichotomous outcomes, we calculated the relative risk (RR) with 95% confidence intervals (CI). To measure continuous outcomes, we calculated the mean difference (MD) with 95% CI. We assessed the heterogeneity of the studies using the I^2^ statistic, with I^2^ > 50% indicates substantial heterogeneity [17]. To ensure the reliability of the results, we used random-effect models for all outcomes and performed a sensitivity analysis using fixed-effect models. We considered a prespecified two-sided *p*-value < 0.05 as statistically significant.

### Subgroup analysis

We conducted subgroup analysis on the primary outcome based on the level of fraction of inspired oxygen, time of publication, and mortality in the control group. We utilized median calculations to establish the cutoff values.

### Sensitivity analysis

We conducted a sensitivity analysis using the following methods: (1) excluding the trial with the highest weight; (2) excluding trials with high risks; (3) employing a fixed-effect model.

### Trial sequential analysis

We carried out trial sequential analysis (TSA 0.9Beta) to prevent an increase in type I error by combining an estimation of information size with an adjusted threshold for statistical significance. We used a two-sided trial sequential analysis to ensure an overall 5% risk of type I error and a power of 80%. Our anticipated intervention effect for the primary outcome was a 25% reduction in relative risk (RR).

## Results

**Fig. 1:**
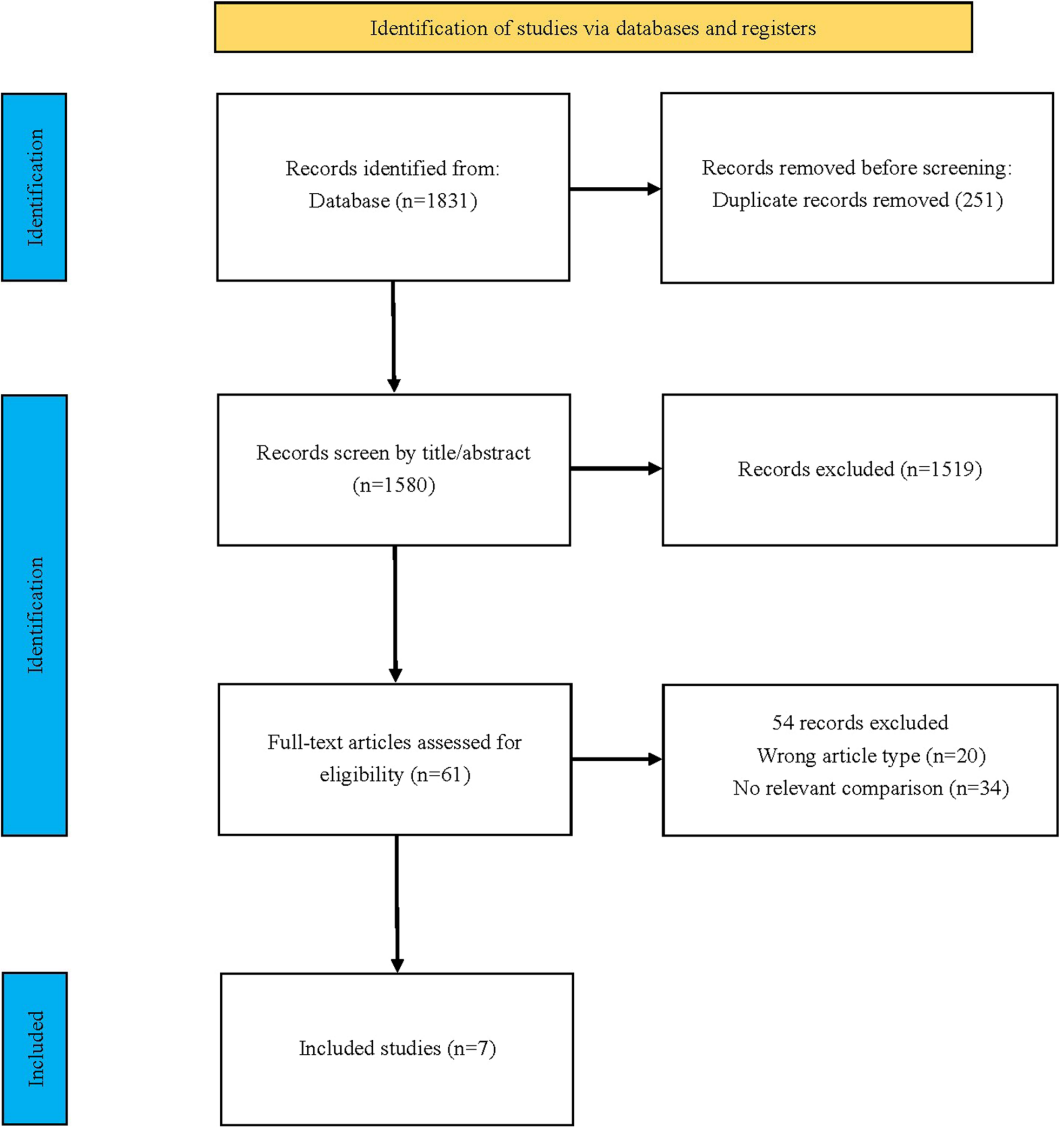
Search strategy and final included and excluded studies

Our search strategy initially identified 1784 records. After removing duplicates, we screened a total of 1580 unique records. Following a thorough evaluation of titles, abstracts, and full texts, we identified 7 trials that satisfied the inclusion criteria for this systematic review [13, 14, 18–22] (Fig. 1).

The characteristics of each trial included in this study are summarized in Table 1. The trials were published between 2006 and 2022, and the sample size ranged from 17 to 789 patients. The average age of participants in each study varied between 59.5 and 67.1 years. All studies were conducted in developed countries.

**Table 1:** Characteristics of included studies

Study	Study region	Liberal group FiO_2_	Conservative group FiO_2_	Delivery method	Participants’ sample size, n	Mean age, years	Female, *n*(%)	Witnessed arrest n(%)	Liberal group, medium baseline SpO_2_ (%)	Conservative group medium baseline SpO_2_ (%)
Kuisma [18]	Finland	1.00	0.33	Mechanical ventilation	28	63.1	5(17.9)	28(100.0)	NA	NA
Young [19]	New Zealand	1	0.4	Ventilated using a self-inflating resuscitation bag	17	66.2	1(5.9)	12(70.6)	95.8%	79.5%
Bray [20]	Australia	NA	NA	Hand ventilation using a bag-valve reservoir	61	62.6	12(19.7)	45(73.8)	NA	98
Jakkula [21]	Finland and Denmark	0.5	0.35	Mechanical ventilation	120	59.5	22(18.3)	120(100.0)	NA	NA
Thomas [22]	United Kingdom	NA	NA	Mechanical ventilation or oropharyngeal airway	35	67.1	10(28.6)	31(88.6)	NA	NA
Bernard [23]	Australia	1	0.7/0.6	Oxygen reservoir bag or mechanical ventilation	425	65.3	100(23.5)	335(78.8)	99	99
Schmidt [24]	Denmark	0.6	0.3	Mechanical ventilation	789	62.5	152(19.3)	672(85.2)	98	98

FiO_2_ Fraction of inspired oxygen

SpO_2_ Arterial saturation of peripheral oxygen

Risk-of-bias assessments are presented in Additional file 1: Fig. S1. Three had low risk of bias [13, 14, 19], one had some concerns [22], and three had high risk of bias [18, 20, 21]. The quality of evidence for the primary outcome was high as evaluated by GRADE (Table 2).

**Table 2:** Quality of evidence

Outcome	Patients	Risk ratio (95% CI)	I^2^(%)	Absolute effect estimates (per 1000)	Quality
Mortality	1451	0.97(0.82, 1.14)	25	− 12 (− 73 to 57)	High
Favorable mRS (0–2)	1163	1.00(0.86, 1.17)	53	0 (− 71 to 87)	Moderate
Favorable CPC (0–2)	1290	1.01(0.90, 1.14)	0	4 (− 43 to 61)	High

*mRS* Modified Rankin scale, *CPC* Cerebral performance category

**Fig. 2:**
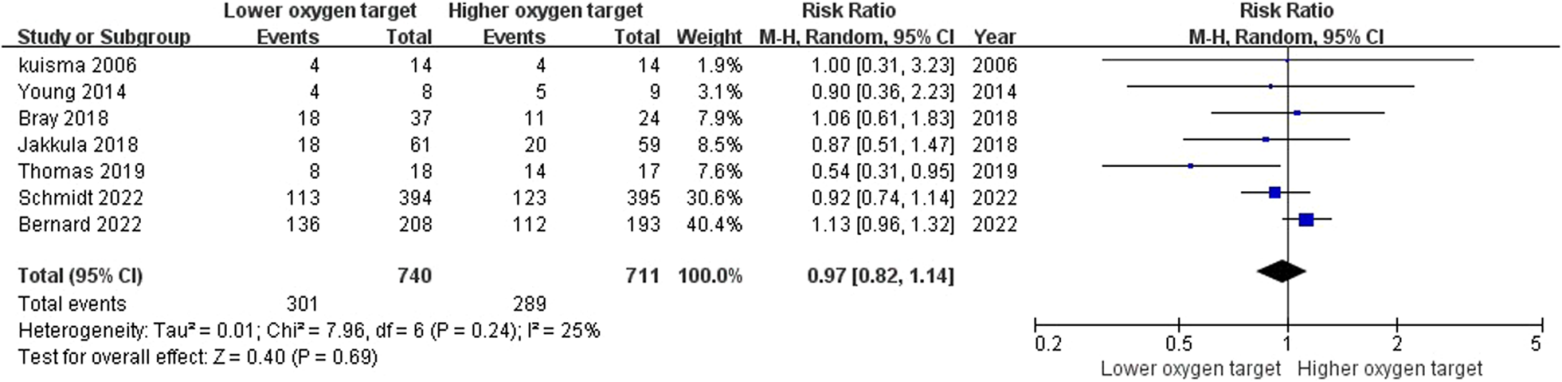
Primary outcome: mortality. Forest plot of comparison: Lower oxygen target versus higher oxygen target for out-of-hospital cardiac arrest, *M–H* Mantel–Haenszel, *CI* Confidence interval, *df* Degrees of freedom

**Fig. 3:**
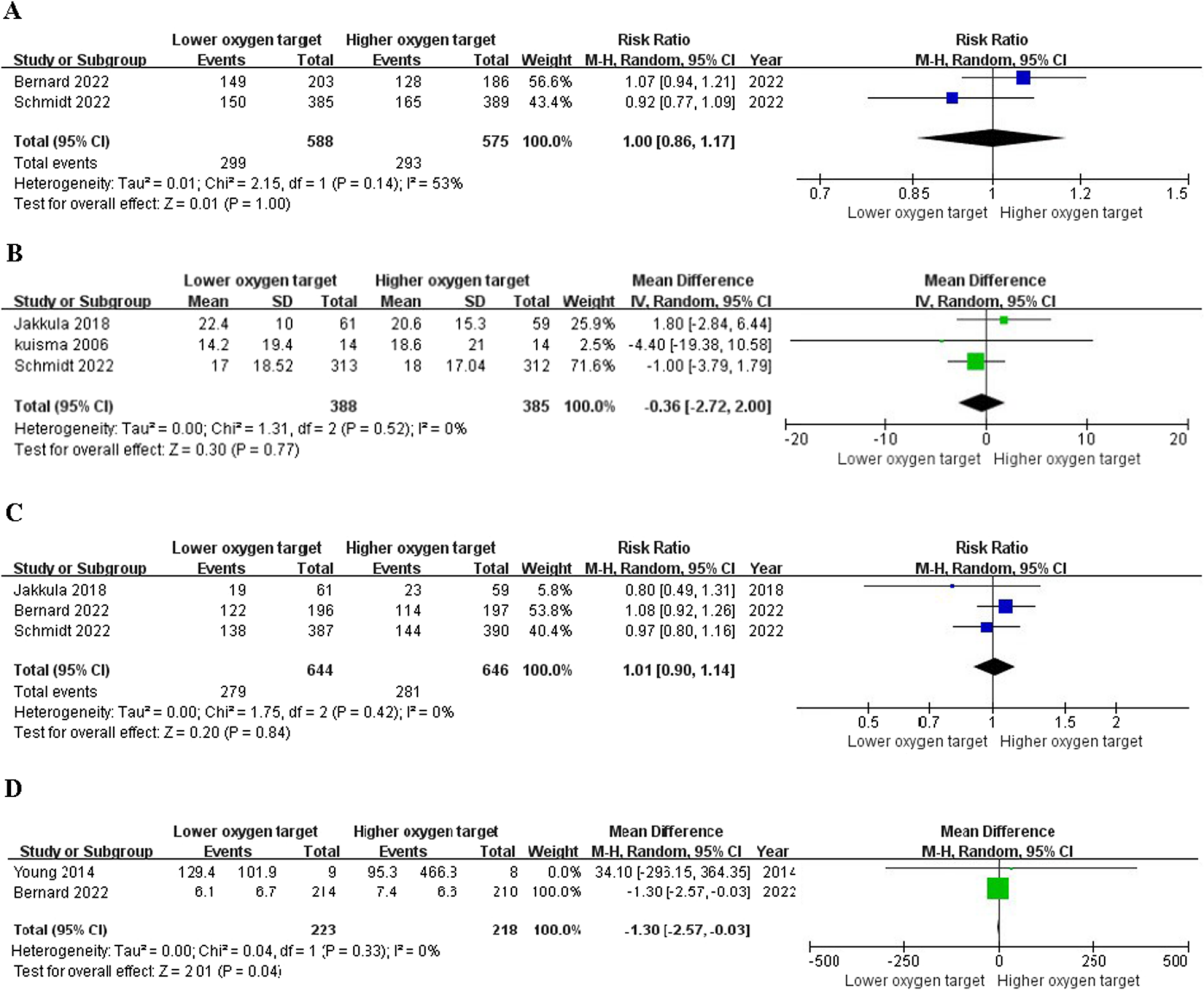
Secondary outcomes. Favorable modified Rankin scale score **A**, Median neuron-specific enolase at 48 h **B**, Favorable Cerebral Performance Category score **C**, Length of hospital stay **D**; *M–H* Mantel–Haenszel, *CI* Confidence interval, *df* Degrees of freedom

Seven trials reported the primary outcome [13, 14, 18–22]. The time points of mortality rates reported in each trial, as well as the time points of mortality rates that we used for our analysis, are demonstrated in Additional file 6: Table S2. Three reported the neuron‑specific enolase at 48 h after OHCA [14, 18, 21], two reported mRS [13, 14], three reported CPC [13, 21], two reported the length of hospital stay [13, 19]. There was no significant difference in mortality between the lower and higher oxygen target groups (RR 0.97, 95%CI 0.82 to 1.14, I^2^ = 25%) (Fig. 2). There was no significant difference in NSE (MD -0.36, 95%CI − 2.72 to 2.00, I^2^ = 0%), favorable mRS (RR 1.00, 95%CI 0.86 to 1.17, I^2^ = 53%) and favorable CPC (RR 1.01, 95%CI 0.90 to 1.14, I^2^ = 0%) (Fig. 3). The lower oxygen target group had a shorter length of hospital stay compared to the higher oxygen target group (MD -1.30, 95%CI − 2.57 to − 0.03) (Fig. 3).

**Fig. 4:**
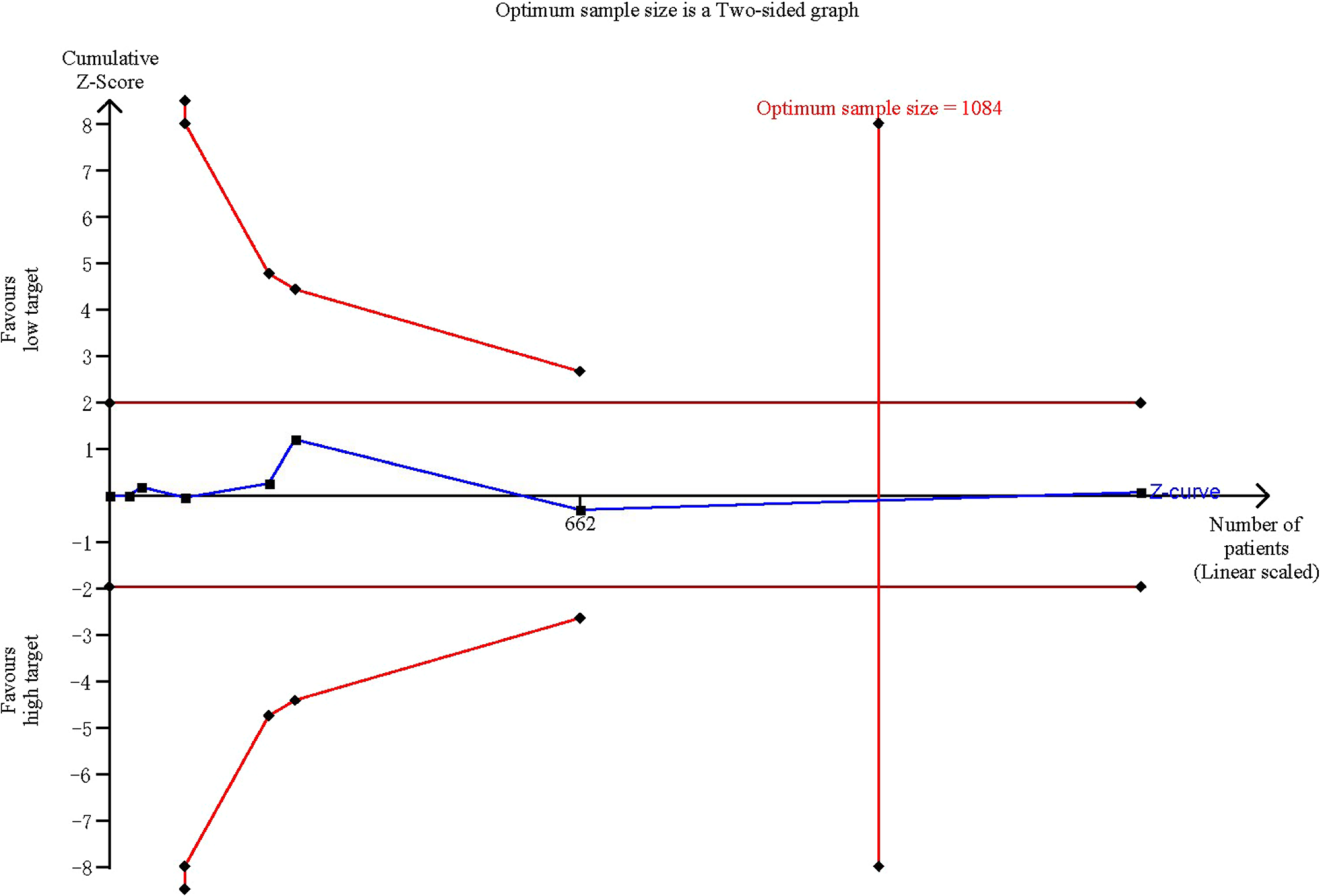
Calculation of optimum sample size. Red vertical line indicates optimum same size (*n* = 1084); blue line indicates Z-curve

The results appeared to be consistent across prespecified subgroups (Additional files 2, 3, 4: Figs. 2 to 4). Additionally, results of all outcomes remained robust to sensitivity analysis (Table 3). Furthermore, the trial sequential analysis of mortality confirmed that the required information size was met (Fig. 4).

**Table 3:** Sensitivity analysis

	Study	RR, 95%CI
Excluding the most weighted trial	Bernard [13]	0.88 (0.74, 1.05)
Excluding trials with high risks	Bray [20], Jakkula [21], Kuisma [18]	0.93 (0.72, 1.19)
Using a fixed-effect model	–	0.99 (0.88, 1.11)

## Discussion

In this meta-analysis of 7 trials with a total of 1475 participants, we found no difference in mortality between lower and higher oxygen targets in patients after OHCA.

Previous meta-analysis of the oxygen target in patients after OHCA showed inconsistent result. Young et al. reported that a lower oxygen target was associated with reduction in mortality at last follow-up compared to higher oxygen target (OR 0.67, 95% CI 0.45 to 0.99) [11], while Holmberg et al. reported no statistical significance (RR 0.97, 95% CI 0.68 to 1.37) [12]. The limitations of these analysis included inclusion of observational studies and inadequate sample size. Our review exclusively included randomized controlled trials, thereby enhancing the reliability of our results. Additionally, we included two large-scale trials focusing on this topic, which had the largest sample size as of February 2023 [13, 14]. The patient sample size in our review is approximately three times larger than that of Young et al. and sixteen times larger than that of Holmberg et al. [11, 12]. Notably, the two latest large-scale trials, which were published in 2022, accounted for 80.7% of the patient sample size (1190/1475).

During our meta-analysis, we did not include any trial that specifically investigated blood oxygen levels above 150 mmHg. According to a network meta-analysis conducted on mechanically ventilated critically ill patients, the liberal goal of maintaining PaO2 levels > 150 mmHg may be inferior to other goals, as indicated by the cumulative ranking curve scores and survival curves [23]. Given the current lack of specific data on higher blood oxygen levels in trials for patients after OHCA, further research that examines the effects of elevated oxygen levels (PaO2 > 150 mmHg) on clinical outcomes is necessary to offer valuable insights and inform clinical decision-making.

The 2017 guideline recommended providing the highest feasible inspired oxygen during resuscitation [4]. However, the administration of oxygen during resuscitation may not necessarily be the same as the oxygen delivered after the return of spontaneous circulation. Studies have recognized this potential distinction and have explored the concept of different oxygen targets during resuscitation and after the return of spontaneous circulation [24].

A review that focused on the optimal combination of airway techniques, oxygenation, and ventilation in patients after OHCA noted that the optimal combination remains uncertain [24]. Existing guidelines and reviews are primarily based on evidence from previous meta-analysis and small-scale trials. Ongoing and recent RCTs are expected to provide additional insights and data. The results of our analysis, which incorporated the most recent trials, indicate that the optimal target should be reconsidered. Moreover, an ongoing trial (NCT05029167) has the potential to either support or refute our conclusions. The trial’s objective is to compare the length of intensive-care-unit stay and mortality between patients receiving PaO2 (98–105 mmHg) with patients receiving PaO2 (68–75 mmHg).

There are several limitations that should be considered. First, the COVID-19 pandemic introduced a risk of bias in the two largest trials included in the meta-analysis. As a result of the pandemic, the trial conducted by Bernard et al. had to be prematurely terminated, which resulted in a smaller sample size than originally planned [13]. Additionally, the trial conducted by Schmidt et al. had evident missing follow-up data for secondary outcomes, although the primary outcomes were complete [14].

Second, there was clinical heterogeneity among trials in our analysis. The trials included in the analysis had different oxygen targets. For instance, higher oxygen target was 98–105 mmHg in the study from Schmidt et al., while it was 150–188 mmHg in the study from Jakkula et al. [14, 21]. Moreover, the duration of oxygenation strategies was different among studies. This was significant because previous studies demonstrated that reduction of the duration of oxygen ventilation after cardiopulmonary resuscitation decreases brain damage [25].

Third, our analysis may lack statistical power to assess neurological impairment due to limited data on the CPC score and mRS, as well as the high statistical heterogeneity observed in the analysis of mRS. The evaluation of neurological impairment is crucial when assessing patients who have survived the acute phase following OHCA [2, 26]. Collection of neurological outcome was heterogeneous among studies. Further research, particularly with standardized neurological outcome measures, is warranted to strengthen and confirm our findings.

Fourth, the generalizability of our findings may be limited as all the trials included in this meta-analysis are primarily from Western countries, particularly Europe and Australia. It is important to note that reducing the upper limits for oxygen saturation is expected to increase the demands on nursing resources. Therefore, these findings may not be applicable to non-western countries that may have limited nursing resources. It is necessary to conduct additional trials in non-western countries to address this limitation.

## Conclusion

Lower oxygen target did not reduce the mortality compared with higher oxygen target in patients after OHCA.

### Supplementary Information

**Additional file 1:** Risk of bias graph.

**Additional file 2:** Subgroup analysis of the level of fraction of inspired oxygen.

**Additional file 3:** Subgroup analysis of the time of publication.

**Additional file 4:** Subgroup analysis of the mortality in control group.

**Additional file 5:** Search strategy.

**Additional file 6:** Time points of mortality rates.

## Data Availability

The datasets used and/or analyzed during the current study are available from the corresponding author on reasonable request.

## Abbreviations

OHCA: Out-of-hospital cardiac arrest
NSE: Neuron‑specific enolase
MRS: Modified Rankin scale
CPC: Cerebral performance category
GRADE: Grading of Recommendation, Assessment, Development and Evaluation
PRISMA: Preferred Reporting Items for Systematic Reviews and Meta-Analysis
CI: Confidence intervals
RR: Relative risk
OR: Odds ratio
MD: Mean difference

## References

[1] Myat A, Song KJ, Rea T (2018). Out-of-hospital cardiac arrest: current concepts. Lancet.

[2] Sandroni C, Cronberg T, Sekhon M (2021). Brain injury after cardiac arrest: pathophysiology, treatment, and prognosis. Intensive Care Med.

[3] Beasley R, Chien J, Douglas J, Eastlake L, Farah C, King G, Moore R, Pilcher J, Richards M, Smith S (2015). Thoracic society of Australia and New Zealand oxygen guidelines for acute oxygen use in adults: ‘Swimming between the flags'. Respirology (Carlton, Vic).

[4] O'Driscoll BR, Howard LS, Earis J, Mak V (2017). BTS guideline for oxygen use in adults in healthcare and emergency settings. Thorax.

[5] Altemeier WA, Sinclair SE (2007). Hyperoxia in the intensive care unit: why more is not always better. Curr Opin Crit Care.

[6] Fracica PJ, Knapp MJ, Piantadosi CA, Takeda K, Fulkerson WJ, Coleman RE, Wolfe WG, Crapo JD (1991). Responses of baboons to prolonged hyperoxia: physiology and qualitative pathology. J Appl Physiol.

[7] Zwemer CF, Whitesall SE, D'Alecy LG (1994). Cardiopulmonary-cerebral resuscitation with 100% oxygen exacerbates neurological dysfunction following nine minutes of normothermic cardiac arrest in dogs. Resuscitation.

[8] Kallet RH, Matthay MA (2013). Hyperoxic acute lung injury. Respir Care.

[9] Suzuki S, Eastwood GM, Peck L, Glassford NJ, Bellomo R (2013). Current oxygen management in mechanically ventilated patients: a prospective observational cohort study. J Crit Care.

[10] Hafner S, Beloncle F, Koch A, Radermacher P, Asfar P (2015). Hyperoxia in intensive care, emergency, and peri-operative medicine: Dr. Jekyll or Mr. Hyde? A 2015 update. Annal Intensive Care.

[11] Young PJ, Bailey M, Bellomo R, Bernard S, Bray J, Jakkula P, Kuisma M, Mackle D, Martin D, Nolan JP (2020). Conservative or liberal oxygen therapy in adults after cardiac arrest: an individual-level patient data meta-analysis of randomised controlled trials. Resuscitation.

[12] Holmberg MJ, Nicholson T, Nolan JP, Schexnayder S, Reynolds J, Nation K, Welsford M, Morley P, Soar J, Berg KM (2020). Oxygenation and ventilation targets after cardiac arrest: a systematic review and meta-analysis. Resuscitation.

[13] Bernard SA, Bray JE, Smith K, Stephenson M, Finn J, Grantham H, Hein C, Masters S, Stub D, Perkins GD (2022). Effect of lower vs higher oxygen saturation targets on survival to hospital discharge among patients resuscitated after out-of-hospital cardiac arrest: the EXACT randomized clinical trial. JAMA.

[14] Schmidt H, Kjaergaard J, Hassager C, Molstrom S, Grand J, Borregaard B, Roelsgaard Obling LE, Veno S, Sarkisian L, Mamaev D (2022). Oxygen targets in comatose survivors of cardiac arrest. N Engl J Med.

[15] Higgins JP, Altman DG, Gøtzsche PC, Jüni P, Moher D, Oxman AD, Savovic J, Schulz KF, Weeks L, Sterne JA (2011). The cochrane collaboration's tool for assessing risk of bias in randomised trials. BMJ.

[16] Guyatt GH, Oxman AD, Vist GE, Kunz R, Falck-Ytter Y, Alonso-Coello P, Schünemann HJ (2008). GRADE: an emerging consensus on rating quality of evidence and strength of recommendations. BMJ.

[17] Higgins JP, Thompson SG (2002). Quantifying heterogeneity in a meta-analysis. Stat Med.

[18] Kuisma M, Boyd J, Voipio V, Alaspää A, Roine RO, Rosenberg P (2006). Comparison of 30 and the 100% inspired oxygen concentrations during early post-resuscitation period: a randomised controlled pilot study. Resuscitation.

[19] Young P, Bailey M, Bellomo R, Bernard S, Dicker B, Freebairn R, Henderson S, Mackle D, McArthur C, McGuinness S (2014). HyperOxic therapy or normoxic therapy after out-of-hospital cardiac arrest (HOT OR NOT): a randomised controlled feasibility trial. Resuscitation.

[20] Bray JE, Hein C, Smith K, Stephenson M, Grantham H, Finn J, Stub D, Cameron P, Bernard S (2018). Oxygen titration after resuscitation from out-of-hospital cardiac arrest: a multi-centre, randomised controlled pilot study (the EXACT pilot trial). Resuscitation.

[21] Jakkula P, Reinikainen M, Hästbacka J, Loisa P, Tiainen M, Pettilä V, Toppila J, Lähde M, Bäcklund M, Okkonen M (2018). Targeting two different levels of both arterial carbon dioxide and arterial oxygen after cardiac arrest and resuscitation: a randomised pilot trial. Intensive Care Med.

[22] Thomas M, Voss S, Benger J, Kirby K, Nolan JP (2019). Cluster randomised comparison of the effectiveness of 100% oxygen versus titrated oxygen in patients with a sustained return of spontaneous circulation following out of hospital cardiac arrest: a feasibility study. PROXY: post ROSC OXYgenation study. BMC Emerg Med.

[23] Zhao X, Xiao H, Dai F, Brodie D, Meng L (2021). Classification and effectiveness of different oxygenation goals in mechanically ventilated critically ill patients: network meta-analysis of randomised controlled trials. Eur Respir J.

[24] Newell C, Grier S, Soar J (2018). Airway and ventilation management during cardiopulmonary resuscitation and after successful resuscitation. Critical Care.

[25] Brücken A, Kaab AB, Kottmann K, Rossaint R, Nolte KW, Weis J, Fries M (2010). Reducing the duration of 100% oxygen ventilation in the early reperfusion period after cardiopulmonary resuscitation decreases striatal brain damage. Resuscitation.

[26] Sandroni C, D'Arrigo S, Cacciola S, Hoedemaekers CWE, Kamps MJA, Oddo M, Taccone FS, Di Rocco A, Meijer FJA, Westhall E (2020). Prediction of poor neurological outcome in comatose survivors of cardiac arrest: a systematic review. Intensive Care Med.

