# Supplementary figures and images for: Lower versus higher oxygen targets for out-of-hospital cardiac arrest: a systematic review and meta-analysis
Source: Crit Care. 2023 Oct 19;27:401. doi: 10.1186/s13054-023-04684-3 (PMC10588244; doi:10.1186/s13054-023-04684-3)

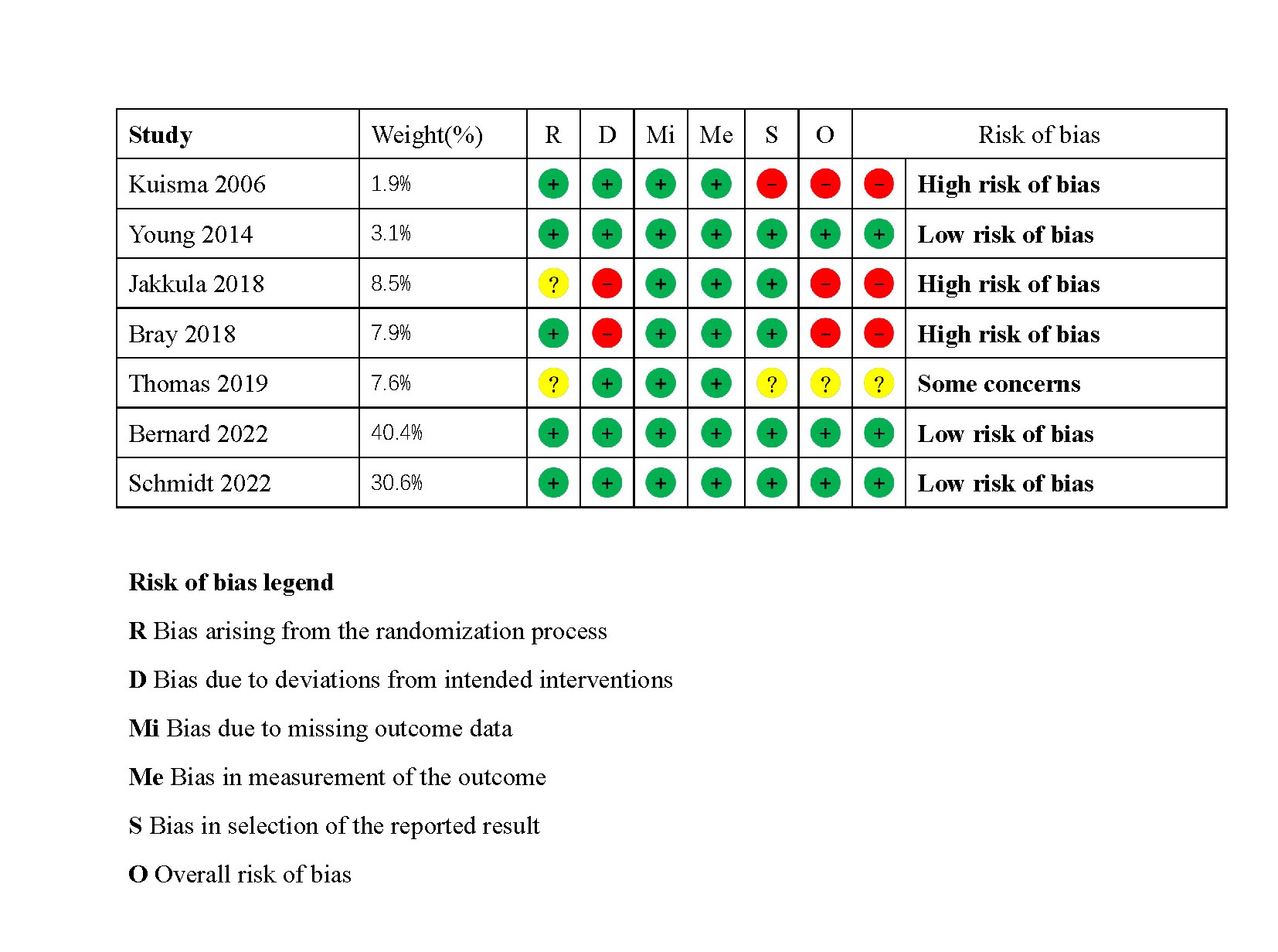

Supplement: Supplementary file 1 — Additional file 1: Risk of bias graph. [file 13054_2023_4684_MOESM1_ESM.jpg]

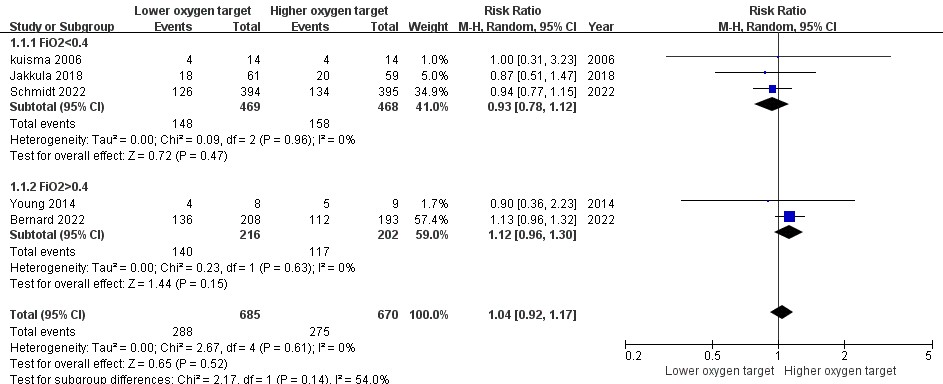

Supplement: Supplementary file 2 — Additional file 2: Subgroup analysis of the level of fraction of inspired oxygen. [file 13054_2023_4684_MOESM2_ESM.jpg]

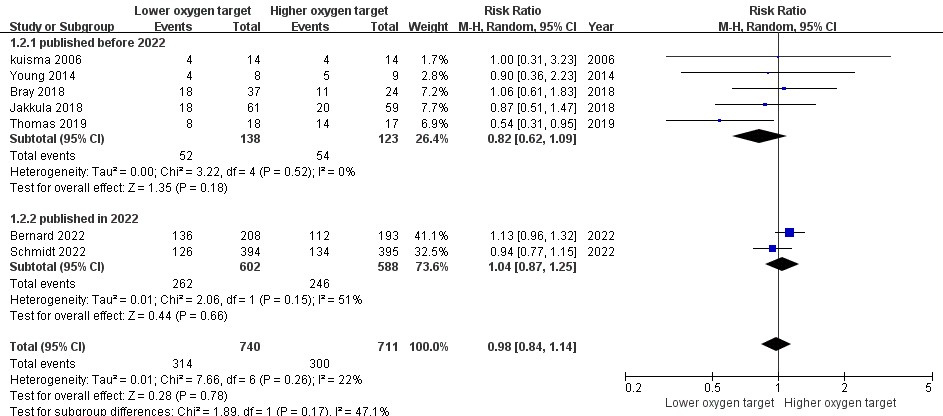

Supplement: Supplementary file 3 — Additional file 3: Subgroup analysis of the time of publication. [file 13054_2023_4684_MOESM3_ESM.jpg]

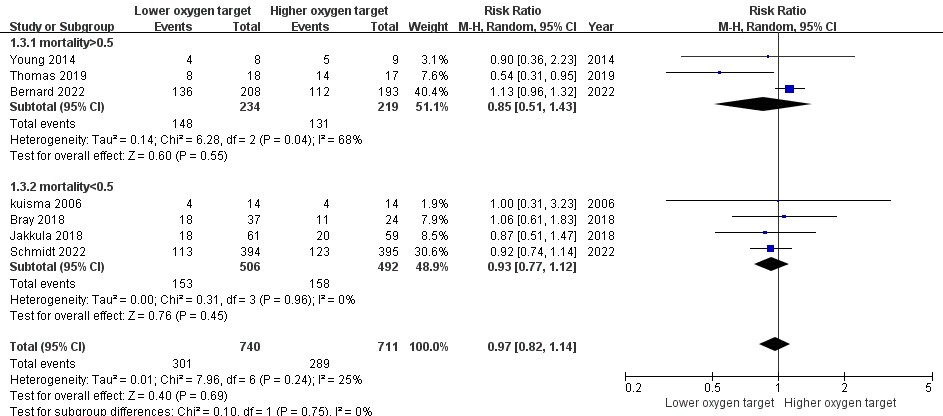

Supplement: Supplementary file 4 — Additional file 4: Subgroup analysis of the mortality in control group. [file 13054_2023_4684_MOESM4_ESM.jpg]
