# Supplementary material for: Lower versus higher oxygen targets for out-of-hospital cardiac arrest: a systematic review and meta-analysis
Source: Crit Care. 2023 Oct 19;27:401. doi: 10.1186/s13054-023-04684-3 (PMC10588244; doi:10.1186/s13054-023-04684-3)
Supplement: Supplementary file 5 — Additional file 5: Search strategy. [file 13054_2023_4684_MOESM5_ESM.docx]

**Supplemental Table 1：Search strategy**

| **Ovid MEDLINE(R)** | | |
| --- | --- | --- |
| 1 | hyperoxia.mp. or exp Hyperoxia/ | 8688 |
| 2 | hyperoxemia.mp. | 307 |
| 3 | hyperoxygenation.mp. | 401 |
| 4 | overoxygenation.mp. | 7 |
| 5 | (permissive adj5 hypoxia).mp. [mp=title, abstract, original title, name of substance word, subject heading word, keyword heading word, protocol supplementary concept word, rare disease supplementary concept word, unique identifier, synonyms] | 33 |
| 6 | (permissive adj5 hypoxemia).mp. [mp=title, abstract, original title, name of substance word, subject heading word, keyword heading word, protocol supplementary concept word, rare disease supplementary concept word, unique identifier, synonyms] | 33 |
| 7 | normoxia.mp. | 9563 |
| 8 | normoxemia.mp. | 182 |
| 9 | liberal*.tw. | 10196 |
| 10 | conservative*.tw. | 119060 |
| 11 | conventional*.tw. | 480640 |
| 12 | 1 or 2 or 3 or 4 or 5 or 6 or 7 or 8 or 9 or 10 or 11 | 623034 |
| 13 | exp Oxygen Inhalation Therapy/ or exp Oxygen/ | 221037 |
| 14 | (supplement* adj5 oxygen*).mp. [mp=title, abstract, original title, name of substance word, subject heading word, keyword heading word, protocol supplementary concept word, rare disease supplementary concept word, unique identifier, synonyms] | 287 |
| 15 | (oxygen* adj5 therap*).mp. [mp=title, abstract, original title, name of substance word, subject heading word, keyword heading word, protocol supplementary concept word, rare disease supplementary concept word, unique identifier, synonyms] | 46 |
| 16 | (inhal* adj5 oxygen*).mp. [mp=title, abstract, original title, name of substance word, subject heading word, keyword heading word,  protocol supplementary concept word, rare disease supplementary concept word, unique identifier, synonyms] | 32 |
| 17 | (inspir* adj5 oxygen*).mp. [mp=title, abstract, original title, name of substance word, subject heading word, keyword heading word,  protocol supplementary concept word, rare disease supplementary concept word, unique identifier, synonyms] | 32 |
| 18 | fio2.mp. | 8053 |
| 19 | 13 or 14 or 15 or 16 or 17 or 18 | 226749 |
| 20 | exp Randomized Controlled Trial/ | 586818 |
| 21 | randomized controlled trial.pt. | 585324 |
| 22 | controlled clinical trial.pt. | 95153 |
| 23 | random*.tw. | 1180371 |
| 24 | placebo.ab. | 215492 |
| 25 | drug therapy.fs. | 2567336 |
| 26 | trial.ab. | 554490 |
| 27 | groups.ab. | 2112099 |
| 28 | 20 or 21 or 22 or 23 or 24 or 25 or 26 or 27 | 5322356 |
| 29 | exp Hospital Mortality/ or mortality.mp. or exp Mortality/ | 1360075 |
| 30 | exp Survival/ or exp Survival Rate/ or survival.mp. or exp Survival Analysis/ | 1369732 |
| 31 | disability.mp. or exp Disability Evaluation/ | 247229 |
| 32 | exp Death/ | 162442 |
| 33 | death*.tw. | 866519 |
| 34 | died.tw. | 245694 |
| 35 | 29 or 30 or 31 or 32 or 33 or 34 | 3104336 |
| 36 | 12 and 19 | 13282 |
| 37 | 28 and 36 | 3452 |
| 38 | 35 and 37 | 802 |
| 39 | exp Exercise/ | 240882 |
| 40 | exercise.tw. | 263461 |
| 41 | training.tw. | 430579 |
| 42 | exp Pregnancy/ | 991662 |
| 43 | exp Obstetrics/ | 24383 |
| 44 | pregna*.tw. | 523451 |
| 45 | obstetric*.tw. | 98164 |
| 46 | mice.tw. | 944299 |
| 47 | mouse.tw. | 646066 |
| 48 | cell*.tw. | 5269896 |
| 49 | vitro.tw. | 1268063 |
| 50 | rodent*.tw. | 114184 |
| 51 | rat.tw. | 854379 |
| 52 | rats.tw. | 840218 |
| 53 | pediatric*.tw. | 309234 |
| 54 | 39 or 40 or 41 or42 or 43 or 44 or 45 or 46 or 47 or 48 or 49 or 50 or 51 or 52 or 53 | 4745 |
| 55 | 38 not 54 | 802 |
| 56 | limit 55 to (animals or pregnancy) | 235 |
| 57 | 55 not 56 | 567 |
| 58 | limit 57 to ("all infant (birth to 23 months)" or "newborn infant (birth to 1 month)" or "infant (1 to 23 months)" or "preschool child (2 to 5 years)" or "child (6 to 12 years)") | 132 |
| 59 | 57 not 58 | 435 |
|  |  |  |
| **Ovid Embase** | | |
| 1 | hyperoxia.mp. or exp hyperoxia/ | 13805 |
| 2 | hyperoxemia.mp. | 496 |
| 3 | hyperoxygenation.mp. | 531 |
| 4 | overoxygenation.mp. | 11 |
| 5 | normoxia.mp. | 15939 |
| 6 | normoxemia.mp. | 263 |
| 7 | liberal*.tw. | 14488 |
| 8 | conservative*.tw. | 190482 |
| 9 | conventional*.tw. | 772296 |
| 10 | exp oxygen therapy/ or exp oxygen/ | 309200 |
| 11 | fio?.tw. | 19213 |
| 12 | randomized controlled trial/ | 761105 |
| 13 | randomized controlled trial.ti. | 71232 |
| 14 | controlled clinical trial.ti. | 9245 |
| 15 | random*.tw. | 1905279 |
| 16 | placebo.ab. | 348320 |
| 17 | drug therapy.fs. | 4246125 |
| 18 | trial.ab. | 941695 |
| 19 | groups.ab. | 3466468 |
| 20 | 12 or 13 or 14 or 15 or 16 or 17 or 18 or 19 | 8674011 |
| 21 | exp mortality/ or exp cardiovascular mortality/ or exp mortality rate/ or mortality risk/ or exp surgical mortality/ or mortality.mp. or exp hospital mortality/ or exp standardized mortality ratio/ | 1837031 |
| 22 | exp survival analysis/ or exp survival rate/ or survival.mp. or exp survival/ or exp median survival time/ or exp cause specific survival/ or exp survival time/ or exp short term survival/ or exp long term survival/ or exp overall survival/ | 2140593 |
| 23 | exp "International Classification of Functioning, Disability and Health"/ or exp Roland Morris disability questionnaire/ or exp disability severity/ or exp language disability/ or exp disability/ or exp Sheehan Disability Scale/ or exp Expanded Disability Status Scale/ or exp ADL disability/ or disability.mp. or exp physical disability/ | 447871 |
| 24 | exp death/ or exp sudden death/ or exp "time of death"/ or exp sudden cardiac death/ | 799942 |
| 25 | death*.tw. | 1423271 |
| 26 | died.tw. | 421650 |
| 27 | exp brain hypoxia/ or exp heart arrest/ | 128854 |
| 28 | 21 or 22 or 23 or 24 or 25 or 26 or 27 | 5180398 |
| 29 | exp exercise/ | 414009 |
| 30 | exercise.tw. | 406787 |
| 31 | training.tw. | 691310 |
| 32 | exp pregnancy/ | 755117 |
| 33 | exp obstetrics/ | 42178 |
| 34 | pregna*.tw. | 747028 |
| 35 | obstetric*.tw. | 159093 |
| 36 | mice.tw. | 1383865 |
| 37 | mouse.tw. | 898649 |
| 38 | cell*.tw. | 7373960 |
| 39 | vitro.tw. | 1731062 |
| 40 | rodent*.tw. | 158173 |
| 41 | rat.tw. | 993964 |
| 42 | rats.tw. | 1073953 |
| 43 | pediatric*.tw. | 556184 |
| 44 | neonat*.tw. | 397786 |
| 45 | newborn/ | 588727 |
| 46 | 29 or 30 or 31 or 32 or 33 or 34 or 35 or 36 or 37 or 38 or 39 or 40 or 41 or 42 or 43 or 44 or 45 | 12329525 |
| 47 | 1 or 2 or 3 or 4 or 5 or 6 or 7 or 8 or 9 | 998158 |
| 48 | 10 or 11 | 322955 |
| 49 | 20 and 28 and 47 and 48 | 2461 |
| 50 | 49 not 46 | 1432 |
| 51 | limit 50 to (animals or animal studies) | 75 |
| 52 | 50 not 51 | 1357 |
| 53 | limit 52 to (embryo <first trimester> or infant <to one year> or preschool child <1 to 6 years> or school child <7 to 12 years>) | 28 |
| 54 | 52 not 53 | 1329 |
| **Cochrane Central Register of Controlled Trials (CENTRAL)** | | |
| #1 | hyperoxia | 812 |
| #2 | MeSH descriptor: [Hyperoxia] explode all trees | 262 |
| #3 | hyperoxemia | 85 |
| #4 | hyperoxygenation | 97 |
| #5 | MeSH descriptor: [Oxygen Inhalation Therapy] explode all trees | 2015 |
| #6 | neonate:ti,ab | 2213 |
| #7 | MeSH descriptor: [Infant, Newborn] explode all trees | 20204 |
| #8 | pediatrics:ti,ab | 2985 |
| #9 | MeSH descriptor: [Pediatrics] explode all trees | 918 |
| #10 | MeSH descriptor: [Pregnancy] explode all trees | 28537 |
| #11 | pregnancy:ti,ab | 50198 |
| #12 | exercise:ti,ab | 97752 |
| #13 | training:ti,ab | 107386 |
| #14 | mice:ti,ab | 4636 |
| #15 | rat:ti,ab | 1688 |
| #16 | MeSH descriptor: [Exercise] explode all trees | 32772 |
| #17 | #6 or #7 or #8 or #9 or #10 or #11 or #12 or #13 or #14 or #15 or #16 | 267004 |
| #18 | (#1 or #2 or #3 or #4) and (#5) | 107 |
| #19 | #18 not #17 | 66 |
