# Supplementary material for: Lower versus higher oxygen targets for out-of-hospital cardiac arrest: a systematic review and meta-analysis
Source: Crit Care. 2023 Oct 19;27:401. doi: 10.1186/s13054-023-04684-3 (PMC10588244; doi:10.1186/s13054-023-04684-3)
Supplement: Supplementary file 6 — Additional file 6: Time points of mortality rates. [file 13054_2023_4684_MOESM6_ESM.docx]

**Supplemental Table 2: Time points of mortality rates**

| Studies | Time points of mortality rates in trials | Time points of mortality rates used for analysis of this study |
| --- | --- | --- |
| Bernard 2022 | In ICU mortality; In hospital mortality; 12 months mortality | In hospital mortality |
| Bray 2018 | In hospital mortality | In hospital mortality |
| Jakkula 2018 | 30 days mortality | 30 days mortality |
| Kuisma 2006 | 48hours mortality; In hospital mortality | In hospital mortality |
| Schmidt 2022 | 90 days mortality; In hospital mortality | 90 days mortality |
| Thomas 2019 | 90 days mortality; In hospital mortality | 90 days mortality |
| Young 2014 | In hospital mortality | In hospital mortality |
